# Isolation and Identification of Polyphenols From Fresh Sweet Sorghum Stems and Their Antibacterial Mechanism Against Foodborne Pathogens

**DOI:** 10.3389/fbioe.2021.770726

**Published:** 2022-02-11

**Authors:** Hao Chen, Yifei Xu, Haoyu Chen, Hao Liu, Qunli Yu, Ling Han

**Affiliations:** ^1^ College of Food Science and Engineering, Gansu Agricultural University, Lanzhou, China; ^2^ Gansu Institute of Food Inspection, Lanzhou, China

**Keywords:** sweet sorghum, polyphenols, isolation and identification, foodborne pathogens, antibacterial mechanism

## Abstract

As a C4 energy crop widely planted all over the world, sweet sorghum is mainly used in sugar making and brewing. Fresh sweet sorghum stalks contain many natural ingredients that have antioxidant properties and can significantly inhibit the growth of foodborne pathogens. In this study, the polyphenols in sweet sorghum were extracted by acid ethanol and ion precipitation, and the types of polyphenols were determined by HPLC-MS. The polyphenol content in fresh sweet sorghum stalks was 5.77 mg/g after process optimization with 18 types of phenolic acids identified. The extract had a total antioxidant capacity of 9.4 μmol Trolox/mL. Polyphenol extract of sweet sorghum displayed antibacterial activity against *Staphylococcus aureus, Escherichia coli, Listeria* spp.*,* and *Salmonella* spp. The extract increased the conductivity of cell suspensions by destroying the membrane structure, resulting in leakage of cell electrolytes. Changes in bacterial morphology and internal structure were indicated. The data describe an optimized process to extract polyphenols from sweet sorghum stalks and the methodology to identify the major components within the extract. The data provide a novel option for the comprehensive utilization of fresh sweet sorghum stalks.

## 1 Introduction

Polyphenols are a natural product in plants that are widely distributed. They can be obtained in large yields (Daglia, 2012). The structures of polyphenols are diverse and include phenolic substances with phenolic hydroxyl groups on a single benzene ring and highly polymerized phenolic substances (Cheynier, 2005). Despite the diversity, they have the same characteristic of potent biological activities, which include antioxidant and antibacterial activities (Crozier et al., 2009). Polyphenols can be classified according to their source, function, and chemical structure. In terms of chemical structure, polyphenols can be simply considered as substances with one or more hydroxyl groups on an aromatic compound. Furthermore, they can be divided into flavone (flavonoids, flavanols, anthocyanins, flavanols, etc.) and flavonoids (coumarin, caffeic acid, and ferulic acid). In addition, some polyphenols can also form complex high molecular weight polymers with polysaccharides, organic acids, proteins, and other substances in plants, such as tannins. Different extraction methods have been used to obtain the phenolic compounds from different kinds of plants. These include organic matter extraction and water extraction. In addition, several physically assisted extraction methods, such as high pressure assisted extraction (Putnik et al., 2016), microwave extraction (Dahmoune et al., 2015), ultrasonic extraction (Kayan et al., 2021), enzyme extraction, and supercritical carbon dioxide extraction (Essien et al., 2020) have been described. These methods have become widely used in the extraction of polyphenols.

Polyphenols can exhibit good antioxidant properties because of their easily reduced phenolic hydroxyl groups. Their antioxidant capacity is related to the group position of phenolic hydroxyl groups. Reflecting the good antioxidant capacity of polyphenols, phenols can reduce some chronic diseases caused by oxidative stress, such as cardiovascular disease and chronic inflammation (Tsao and Rong, 2010). In addition to their antioxidant capacity, many phenolic compounds can also exhibit antibacterial activity (Sikkema et al., 1995). The complete details of the antibacterial mechanism of phenolic compounds remain unclear. However, it is known that phenolic substances can interact with the cell membrane structure, resulting in irreversible damage that inhibits cell growth and reproduction (Chirico and Gramatica, 2011). Polyphenols can reportedly change the permeability of cell membranes and lead to changes in various cellular functions. Polyphenols can also combine with cell enzymes, resulting in altered cell wall stiffness (Gokhale and Kulkarni, 2000). Phenolic acids destroy the integrity of cell membranes, resulting in the leakage of intracellular substances (Borges et al., 2013). Flavonoids can inhibit energy metabolism in cells and inhibit essential enzymes required for DNA and RNA replication, resulting in cell death (Hiroyuki et al., 1998).

These substances are stored in roots, stems, leaves, fruits, and seeds of many plants, including sweet sorghum. This gluten-free grain is the fifth most-harvested crop globally. It is an economically important agricultural crop with highly efficient photosynthetic activity. It is the main food source in Asia and Africa. Sweet sorghum has a high biological yield and high fermented sugar content (Petr et al., 2003), excellent salt tolerance and drought resistance, and can be planted in semi-arid areas.

Sweet sorghum is planted worldwide as a bioenergy crop (Naeini et al., 2016). Therefore, as a non-food crop, sweet sorghum has great potential for development. However, sweet sorghum is mainly used as a raw material for a single purpose, and research has mainly focused on its sugar or cellulose components (Yu et al., 2012). The straw by-product is seldom used and is typically stripped-off and discarded. Sweet sorghum stalk anthocyanins, phenolic acids, and sterols are good sources of phytochemicals. It is also a potential source of antioxidants (Yang et al., 2015). The stems are rich in phenolic compounds, which may play a role in the prevention of some chronic diseases caused by oxidative stress (Soltani et al., 2021).

The roles of polyphenols as antioxidants, preservatives, and bacteriostatic agents has been widely studied in recent years, and microbial resistance to antibiotics has become an active research area. However, sweet sorghum, as a C4 energy compound widely planted all over the world, is rarely studied in this field. In this context, exploration of the potential value of polyphenol extract from fresh sweet sorghum stem as a natural antioxidant and antibacterial agent is warranted.

## 2 Materials and Methods

### 2.1 Chemicals and Materials

Folin phenol reagent, ethanol, ethyl acetate, hydrochloric acid, AlCl_3_, ZnCl_2_, beef extract, peptone, NaCl, agar, phosphate buffered saline (PBS), and Coomassie brilliant blue were obtained as analytically pure products from Beijing Solabao Biology Co., Ltd. Sweet sorghum (BJ0602) was grown in saline soil in Wuwei City (longitude: 102.634; Latitude: 37.929), Gansu Province, China, at a site at the Radiation Mutation Engineering Laboratory. It was planted in early May 2020 and harvested on October 15, 2020. The total fermented sugar content of sweet sorghum was relatively high, accounting for approximately 12–13% of the total sugar content. To make complete use of sweet sorghum, straw residue was used to extract phenolic acids.

### 2.2 Extraction of Polyphenols From Fresh Sweet Sorghum Stalks

Polyphenol extraction from fresh sweet sorghum stalks was performed as previously described (Chen et al., 2021) with some modifications. Briefly, the newly harvested sweet sorghum stems were collected from the sweet sorghum planting base, washed, and then cut into small sections of approximately 10 cm using a juicer. After juicing, the fresh sweet sorghum straw juice residue mixture was vacuum filtered with medium-speed qualitative filter paper to remove the residue. The sample served as the control for the determination of total phenol content and total antioxidant capacity. One hundred grams of fresh sweet sorghum stalk juice residue mixture was added to 500 ml of acid ethanol with an ethanol content of 60% (1.6 mol/L HCl, volume percentage 2%) and the pH was adjusted to 6.0 with NaOH buffer. The mixture was stirred and extracted in a 600 W ultrasonic water bath.

After extraction, the extract and residue were separated using medium-speed qualitative filter paper. The residue was collected and three cycles of washing and suction were performed to completely wash the contents. The dilute solution of polyphenol crude extract (1 L) received 50 g AlCl_3_ + ZnCl_2_ composite precipitant (mass ratio AlCl_3_: ZnCl_2_ = 1:2; mass ratio of precipitant: sweet sorghum stalk = 1:20) at room temperature to precipitate polyphenol at an adjusted pH of 6.0, using 1 mol/L NaHCO_3_. After allowing the sample to stand for 30 min, it was centrifuged at room temperature and 6,000 rpm for 15 min. The supernatant was discarded to obtain a complex of polyphenols and precipitate.

A total of 100 g of the complex of polyphenol and precipitant was washed by addition to 500 ml of 60% acid ethanol. After the extract and wash solution were fully mixed, they were centrifuged at 6,000 rpm for 15 min at room temperature. The supernatant was removed and washing was repeated. The procedure was repeated two more times. Following the final wash, 500 ml of 1 mol/L HCl solution was added to the precipitate to completely dissolve it. The same volume of ethyl acetate was added to the solution for static extraction. Each extraction was performed three times for 15 min each.

The extract (500 ml) was placed in a round-bottom flask. Ethyl acetate was recovered by rotary evaporation at 40°C. A polyphenol concentrate was obtained. The concentrate was dried in an aluminum box at room temperature to completely volatilize the ethyl acetate. Subsequently, a small amount of deionized water was added to redissolve it to obtain the polyphenol purification concentrate. The concentrated solution was frozen at-80°C for 18 h and then lyophilized under vacuum at -60°C for 36 h to obtain fresh sweet sorghum stem polyphenol lyophilized powder.

### 2.3 Optimization of Extraction Technology of Polyphenols From Sweet Sorghum Stalks

#### 2.3.1 Standard Curve

The standard curve was determined using the classical and widely used Folin method (Li et al., 2008). The gallic acid standard was prepared at 0.01, 0.02, 0.03, 0.04 and 0.05 mg/ml. One milliliter of each solution was fully reacted with 0.25 ml of Folin reagent for 5 min in the dark at 25°C. Further, 1 ml of Na_2_CO_3_ solution was added. After the mixture was fully mixed, it was heated in a water bath at 50°C for 10 min, cooled to 25°C, and the absorbance was measured at 760 nm.

#### 2.3.2 Determination of Polyphenol Extraction Rate

The crude polyphenol extract was extracted, and the polyphenol content was determined using the standard curve described above. Three measurements were made and the average value was reported. The polyphenol content was calculated using the linear regression equation:

$$\text{Extraction}\;\text{rate}\left(\text{mg}.\;{\text{g}}^{-1}\right)=\left(\text{C}\times \text{V}\times \text{n}\right)/\text{m},$$

Where C is the polyphenol concentration in mg/mL, V is the volume of crude extract (ml), n is the dilution ratio, and m is the weight of the raw material (g).

#### 2.3.3 Single Factor Experiment

The effects on the extraction rate were determined for acidic ethanol (40, 50, 60, 70, and 80%; 1.6 mol/L HCl, volume percentage 2%), liquid-solid ratio (5:1, 10:1, 15:1, 20:1, and 25:1; mL: g), ultrasonic time (30, 60, 90, 120, and 150 min), and ultrasonic temperature (30, 40, 50, 60, and 70 °C).

#### 2.3.4 Response Surface Optimization

In each single factor experiment, the top three high levels of extraction rate were selected. In the whole process of polyphenol extraction, the response surface design method of four factors and three levels was adopted to systematically optimize the above four conditions (Zhang et al., 2009).

### 2.4 Analysis of Polyphenol Extracts From Sweet Sorghum Stalks

The liquid chromatography-mass spectrometry (LC-MS) method used was slightly modified according to Wei et al. (2013) and Garcia (2011). LC-MS was performed using an Ultimate 3000 LC device and Q Exactive HF (Thermo Fisher Scientific) equipped with a newly activated C18 column (Zorbax eclipse C18, 2.0 μm^∗^2.3^∗^120 mm), and the extract analysis platform of Qingdao Kechuang Quality Detection Co., Ltd. to determine the types and contents of the main phenolic compounds in sweet sorghum extract. The system operated with a column temperature of 30°C, flow velocity of 0.3 ml/min, mobile phase A of water and 0.1% formic acid, and mobile phase B of acetonitrile. Two microliters of sample were used for each determination. The sample injection temperature of the automatic sampler was set to 4°C, and the mobile phase was 0.1% formic acid and H_2_O (A) and acetonitrile (B). The gradient used was 95% solution A from 0 to 2 min, followed by the decrease of A from 95% at 2 min to 5% at 20 min, followed by the flow of 5% solution A at 300 μL/min for 5 min. The mass spectrometry conditions were as follows: heater temperature 325°C, sheath gas velocity 45 arb, auxiliary gas flow rate of 15 arb, purge gas flow rate of one arb, electrospray voltage of 3.5 KV, capillary temperature of 330°C, and S-Lens RF level of 55%. Scanning was done in the full scan mode from 100 to 1,500 m/z). Data-dependent secondary mass spectrometry (dd-MS2, TopN = 10) was performed at a resolution of 120,000 (primary MS) and 60,000 (secondary MS). The collision mode was high-energy collision dissociation.

### 2.5 Determination of Total Phenol Content and Total Antioxidant Capacity

Briefly, 10 μL of polyphenol sample solution was repeatedly mixed with the corresponding liquid in the total phenol content kit (Suzhou Keming Biotechnology Co., Ltd. Suzhou, China), measured at 760 nm, and the total phenol content of the sample was converted according to the standard curve. The results are expressed as mg/g sample. The antioxidant capacity of the samples was determined using the Total Antioxidant Capacity kit (Suzhou Keming Biotechnology Co., Ltd. Suzhou, China). After the sample was fully mixed with the reagent, the absorbance was measured at 593 nm. The T-AOC of the extract was expressed as μmol Trolox/mL.

### 2.6 Antibacterial Activity of Polyphenols From Sweet Sorghum Stalks

#### 2.6.1 Microbial Cultivation

*Staphylococcus aureus* CMCC 26003, *Listeria monocytogenes* ATCC 19114, *Escherichia coli* CMCC 4410, and *Salmonella* Typhimurium ATCC 14028 were obtained from the China Medical Culture Center. The antibacterial activity of polyphenol extracts from sweet sorghum was studied. Each strain was inoculated in 25 ml nutrient broth medium (peptone 10 g/L, beef extract 3 g/L, sodium chloride 5.0 g/L), incubated at 37 °C for 8 h in a ZQLY-180 N rotary incubator (Shanghai Zhichu Co., Ltd.) as previously described (Jy et al., 2004). The optical density at 600 nm (OD_600_) of each strain was adjusted to 1.0. The bacterial suspension (100 μL) was centrifuged at 12,000 rpm for 15 min to obtain the precipitate. The cells of each strain were resuspended in 10 μL of different concentrations (20 g/L) of sweet sorghum phenolic compounds extract and treated for 10 min. Ten microliters of PBS buffer was added to the blank control group of each strain, fully suspended, and maintained for 10 min.

#### 2.6.2 Flow Cytometry

Annexin V-FITC/PI staining (BD Pharmingen, San Diego, CA, United States) was used to assess the degree of apoptosis. The treated cells were fully mixed with 500 μL buffer and reacted with annexin V conjugated to fluorescein isothiocyanate (FITC) and with propidium iodide (PI) for 15 min at 25°C in the dark. Annexin V-positive cells were regarded as apoptotic cells (Xia et al., 2019).

#### 2.6.3 Permeability of Bacterial Membrane

Conductivity was determined by a modification of a previously described method (Lee et al., 1998). After incubation at 37°C overnight, the bacteria were centrifuged at 10,000 rpm for 10 min at room temperature. The collected bacteria were washed with 10 mM PBS (pH 7.0) three times and diluted to approximately 10^5^ colony forming units (CFU)/mL with PBS. The polyphenol extract of sweet sorghum was added to the bacterial cell suspension until the final concentration was 20 mg/ml. PBS buffer with the same volume as the polyphenol extract solution was added to the blank control group. The samples were mixed and incubated at 37°C with shaking (150 rpm at room temperature). The conductivity of the cell culture medium was measured at 0, 1, 2, 3, 4, 5, 6, and 7 h.

#### 2.6.4 Bacterial Protein and Nucleic Acid Leakage

Protein leakage was checked following a previously described method with modifications (Zhao et al., 2017). Bacterial suspension (100 µL) was treated with sweet sorghum phenolic extract and then centrifuged at 12,000 rpm for 15 min at room temperature. The supernatant (20 μL) and 200 μL Coomassie Brilliant Blue G-250 solution diluted with deionized water was mixed at 25°C for 5 min. A fluorescent plate reader was used to measure the absorption of the mixture at 595 nm of the sweet sorghum phenolic compound extract (100 μL). The treated bacterial suspension was then centrifuged at 12,000 rpm for 15 min at room temperature. A 0.22 μM pore size sterile microporous membrane filter was used to filter the supernatant. The remaining liquid was diluted 100-fold and the nucleic acid concentration was measured as described above.

### 2.7 Statistical Analysis

All tests were repeated three times in parallel. All data are expressed as the mean ± standard deviation (SD; n = 3). The experimental results were evaluated by one-way ANOVA. Significant differences between the means were analyzed by Tukey’s test (*p* < 0.001).

## 3 Results

### 3.1 Optimization of Polyphenol Extraction Process From Sweet Sorghum Stalks

#### 3.1.1 Single Factor Test

**FIGURE 1:**
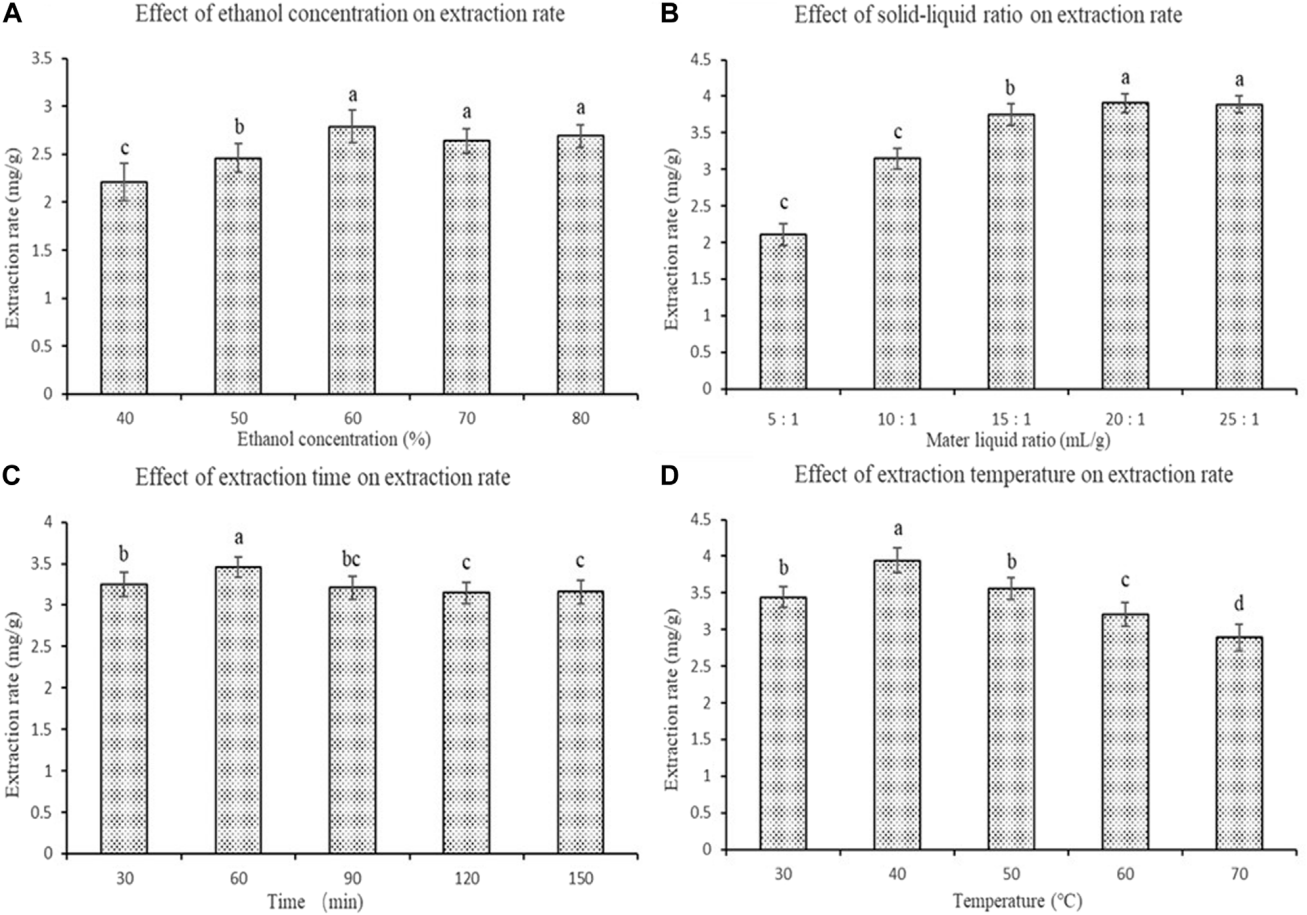
Effects of four single factors on the extraction rate of polyphenols from sweet sorghum **(A)** is the ethanol concentration **(B)** is the material liquid ratio **(C)** is the extraction time and **(D)** is the extraction temperature. The data are expressed as the mean ± standard deviation. One-way ANOVA (*p* < 0.001) and Dunnett’s posttest were used for statistical analysis. Different lowercase letters in the figure indicate differences in significance.

First, we studied the extraction rate of polyphenols from fresh sweet sorghum stems using a single factor experiment. The extraction rate of polyphenols from fresh stems of sweet sorghum first increased and then decreased with increasing ethanol concentration. Ultimately it became stable (Figure 1A). However, when the concentration of ethanol was 60%, the polyphenol content in the extract was the highest. The extraction rate was significantly higher than that of ethanol when the concentration of ethanol was 50% (*p* < 0.05). At the same time, with an increase in the solid-liquid ratio within a certain range, the extraction of polyphenols from fresh sweet sorghum stems increased first and then remained unchanged. The findings indicated that a certain volume of acid ethanol solution promoted the dissolution of polyphenols in the extraction process. When the solid-liquid ratio was 20:1 ml/g, the polyphenol extraction rate of fresh sweet sorghum stalk was the highest (Figure 1B).

During the entire extraction process, with progressively longer ultrasonic treatment times, the polyphenol extraction rate of fresh sweet sorghum stalk first increased and then decreased. When the ultrasonic treatment time was 60 min, the polyphenol extraction rate of fresh sweet sorghum stems was the highest (Figure 1C). In addition, the effect of temperature on the extraction rate of polyphenols from fresh sweet sorghum stems was investigated. During the entire extraction process, with an increase in the extraction temperature, the extraction rate first increased and then decreased. The extraction rate was the highest at 40 °C (Figure 1D).

#### 3.1.2 Analysis of Response Surface Test

Using the basic principle of response surface test design, to study the effect of the test on the extraction rate we used a four-factor and three-level experimental design. The influencing factors were ethanol concentration (A), liquid-solid ratio (B), ultrasonic time (C), and ultrasonic temperature (D). Tables 1 and 2 show the experimental design, design results, and analysis of variance of the response surface test. In the response surface test design table, 0 (medium level) represents the optimal extraction conditions obtained through the single factor test,-1 (low level) represents the extraction conditions on the left side of the optimal conditions, and 1 (high level) represents the extraction conditions on the right side of the optimal conditions.

**TABLE 1:** Response surface experimental design and results.

Serial number	*A* ethanol concentration (%)	*B* liquid to material ratio (mL/g)	*C* ultrasonic time (min)	*D Ultrasonic temperature* (*°C*)	*Y* extraction rate (mg/g)
1	−1	0	1	0	3.77
2	0	0	0	0	5.37
3	0	0	1	1	4.36
4	0	0	0	0	5.42
5	0	−1	0	1	3.54
6	−1	0	−1	0	4.17
7	0	0	0	0	5.77
8	0	1	0	−1	4.36
9	1	0	−1	0	3.65
10	1	0	0	1	4.14
11	−1	0	0	−1	3.89
12	0	1	1	0	4.15
13	0	−1	0	−1	3.98
14	1	0	1	0	4.33
15	0	0	0	0	5.17
16	0	1	−1	0	4.06
17	0	1	0	1	3.76
18	0	0	−1	−1	4.15
19	1	−1	0	0	4.33
20	0	0	0	0	5.68
21	0	−1	−1	0	3.89
22	0	0	1	−1	3.67
23	−1	1	0	0	4.54
24	0	−1	1	0	3.64
25	−1	−1	0	0	3.84
26	0	0	−1	1	3.56
27	1	1	0	0	4.08
28	−1	0	0	1	4.09
29	1	0	0	−1	4.57

**TABLE 2:** Analysis of variance of regression equation.

Source of variation	Freedom	Sum of squares	Mean square	*F*	P	Significance
Model	14	9.215546	0.659874	25.05456	<0.0001	**
A	1	0.045479	0.079465	2.815645	0.1164	
B	1	0.256413	0.251546	9.515454	0.0084	**
C	1	0.0003	0.0003	0.011456	0.9164	
D	1	0.099011	0.099008	3.848,465	0.0725	
AB	1	0.15987	0.1684	6.404265	0.0251	*
AC	1	0.156987	0.156984	5.944416	0.0254	*
AD	1	0.094026	0.093115	3.545,713	0.0887	
BC	1	0.0731	0.0726	2.785412	0.1187	
BD	1	0.018225	0.018354	0.695461	0.4175	
CD	1	0.265,645	0.265,632	10.11145	0.0059	**
A2	1	2.156987	2.153,026	82.04545	<0.0001	**
B2	1	2.564566	2.931564	111.7124	<0.0001	**
C2	1	4.060156	4.061065	154.6545	<0.0001	**
D2	1	3.26978	3.26846	124.5324	<0.0001	**
Residual	14	0.369879	0.026248			
Misfit term	10	0.16547	0.010546	0.156489	0.9921	
Pure error	4	0.25467	0.06617			
Sum	28	9.589746				

* * (*p* < 0.01) * The difference was significant (*p* < 0.05).

**FIGURE 2:**
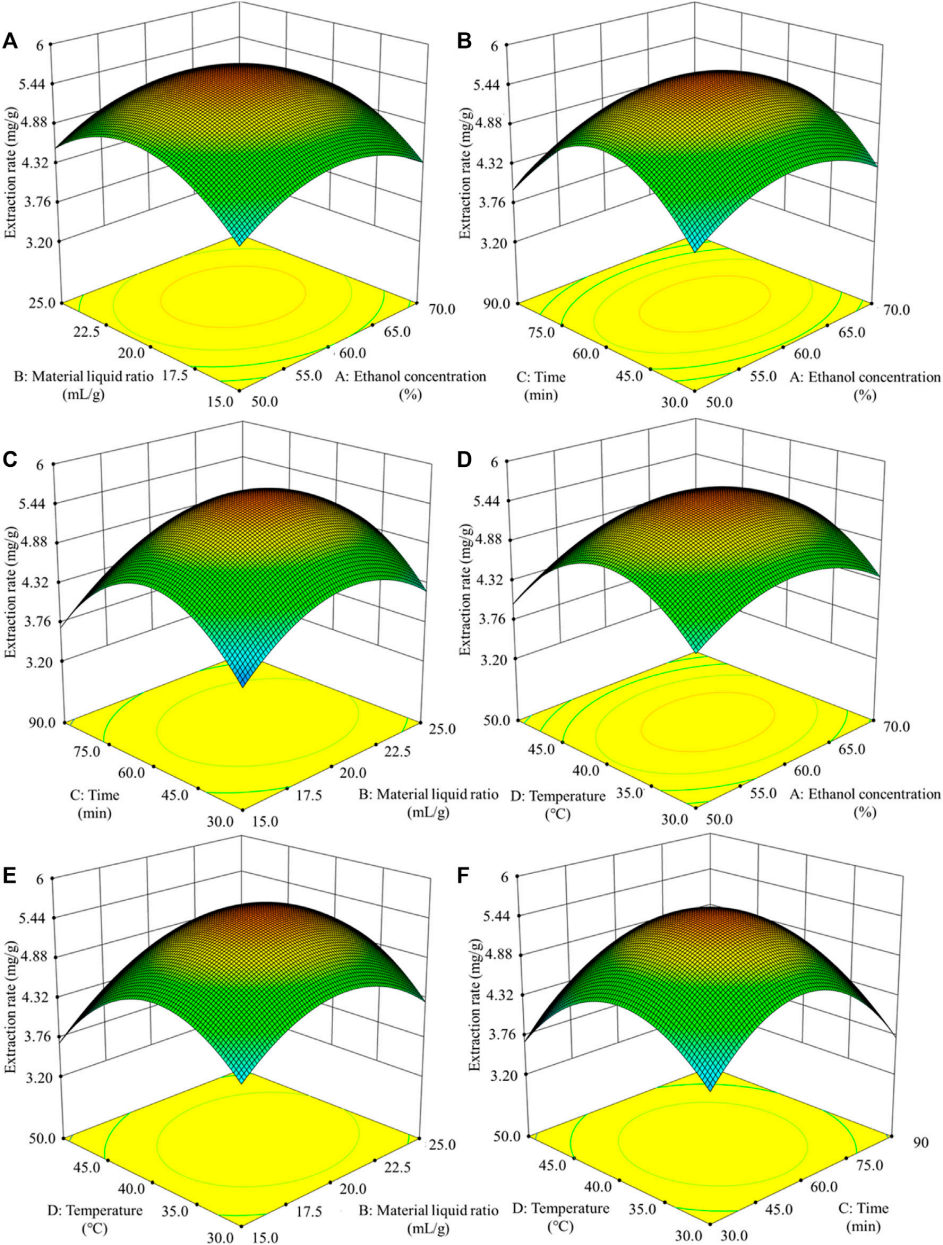
Response surface curve of the influence of the interaction of various factors on the extraction rate of polyphenols **(A)** displays the influence of solid-liquid ratio and ethanol concentration on extraction rate **(B)** displays the influence of extraction time and ethanol concentration on extraction rate **(C)** displays the influence of extraction time and solid-liquid ratio on extraction rate **(D)** displays the influence of extraction temperature and ethanol concentration on extraction rate **(E)** displays the influence of extraction temperature and solid-liquid ratio on extraction rate **(F)** displays the influence of extraction temperature and extraction time on extraction rate.

The response surface diagram of the effects of the four different factors on the extraction rate of polyphenols from sweet sorghum is shown in Figure 2. The diagram mainly reflects the interaction of ethanol concentration, liquid-solid ratio, ultrasonic time, and ultrasonic temperature. The steeper and more inclined the slope of the response surface graph, the stronger the interaction between the two factors. As seen from the figure, the interaction between the ultrasonic temperature and ultrasonic time was very significant. The interactions between ethanol concentration and liquid-solid ratio, ethanol concentration, and ultrasonic time were also very significant.

### 3.2 Total Phenol Content, Phenolic Components, and Antioxidant Capacity

**FIGURE 3:**
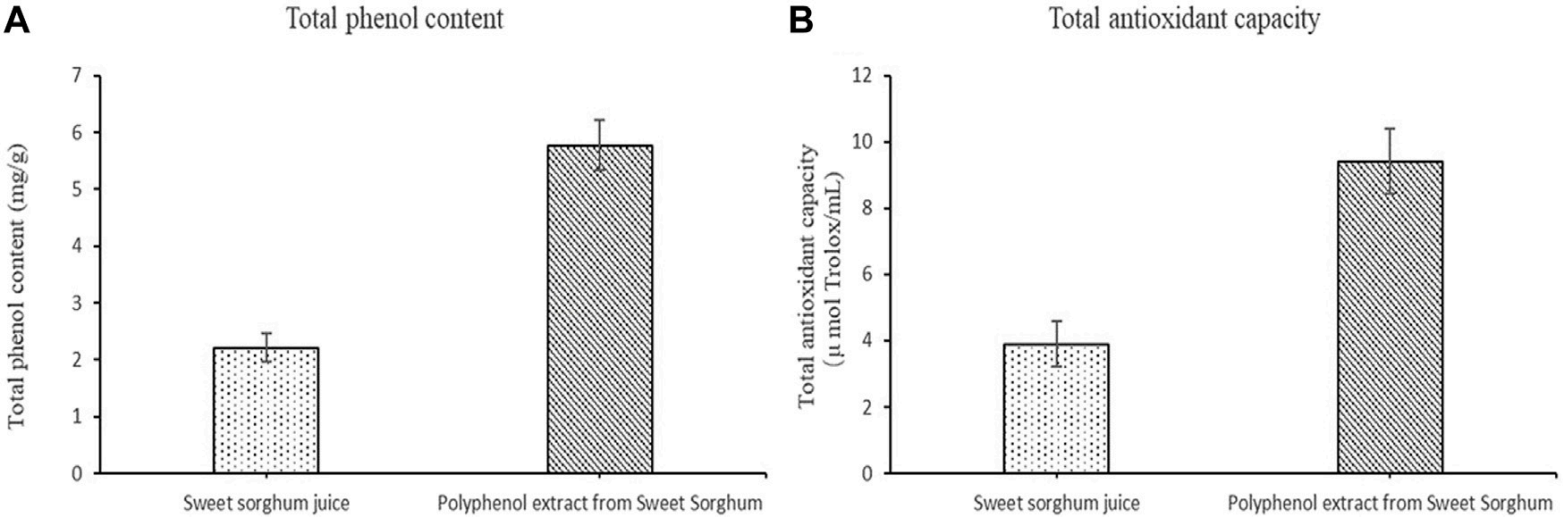
Total phenol content and total antioxidant capacity of sweet sorghum juice before and after extraction **(A)** is the comparison of total phenol content before and after extraction **(B)** is the comparison of total antioxidant capacity before and after extraction. The data are expressed as mean ± standard deviation. One-way ANOVA (*p* < 0.001) and Dunnett’s posttest were used for statistical analysis.

**FIGURE 4:**
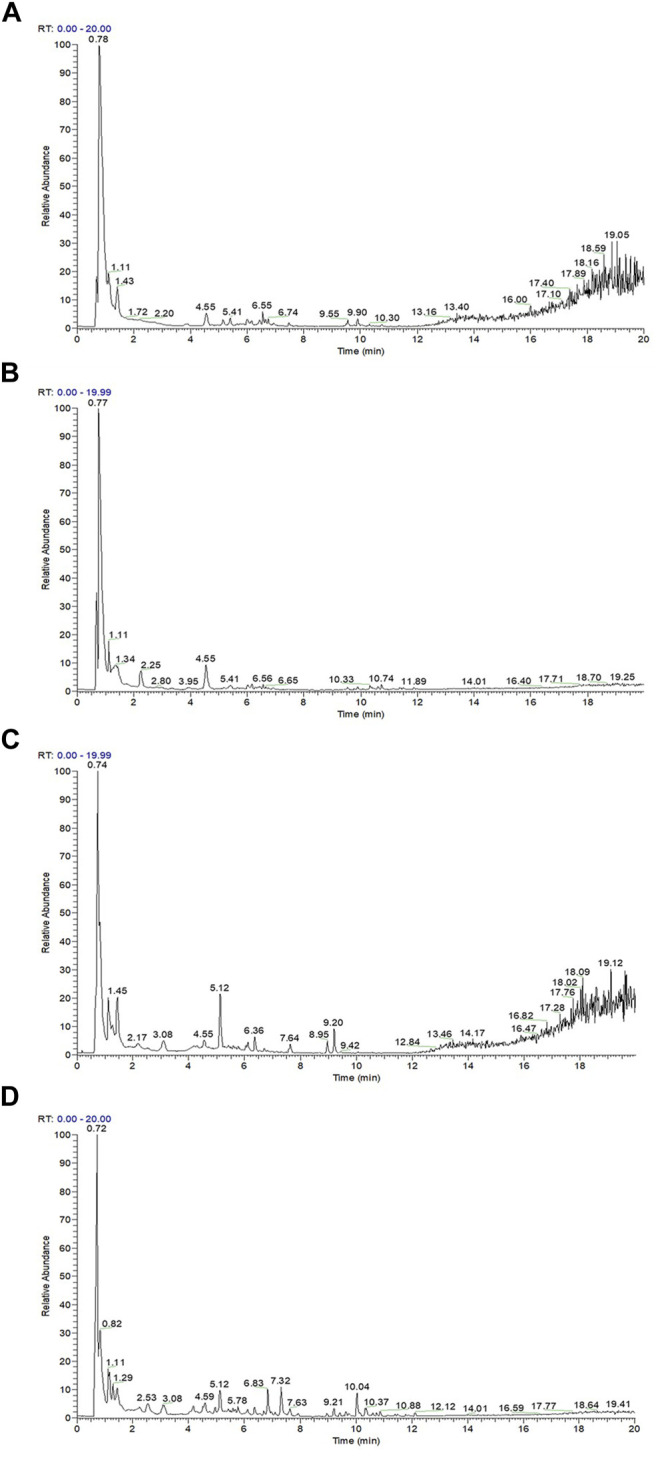
Determination of positive and negative ion current patterns of sweet sorghum juice before and after extraction by liquid chromatography-mass spectrometry **(A)** is the positive ion flow diagram in sweet sorghum juice before extraction **(B)** is the negative ion flow diagram in sweet sorghum juice before extraction **(C)** is the positive ion flow diagram in the extracted sweet sorghum polyphenol extract **(D)** is the negative ion flow diagram in the extracted sweet sorghum polyphenol extract.

The total phenol content, total antioxidant capacity, polyphenol species, and polyphenol content in the polyphenol extract of fresh sweet sorghum stem were determined by spectrophotometry and LC-MS. The total phenol content in sweet sorghum juice was 2.21 mg/g after extraction and purification. The total phenol content was 5.77 mg/g (Figure 3A). The total antioxidant capacity of sweet sorghum juice and sweet sorghum polyphenol extracts before and after extraction were also determined. The total antioxidant capacity was 3.9 μM Trolox/mL before extraction and 9.4 μM Trolox/mL after extraction and purification (Figure 3B). Figure 4 shows the LC-MS total ion flow diagram of sweet sorghum juice and sweet sorghum polyphenol extract. Figure 4A shows the positive ion flow diagram of sweet sorghum juice before extraction. Figure 4B shows the negative ion flow diagram in sweet sorghum juice before extraction. Figure 4C shows the positive ion flow diagram in the extracted sweet sorghum polyphenol extract. Finally, Figure 4D shows the negative ion flow diagram in the extracted sweet sorghum polyphenol extract.

The detailed contents of phenolic acids in sweet sorghum juice and sweet sorghum polyphenol extracts are summarized in Table 3. The most abundant phenolic acid in raw juice was betaine (51.207 μg/ml), *p*-coumaraldehyde (22.448 μg/ml), guanosine (14.398 μg/ml), pseudouridine (14.221 μg/ml), 2,2′-methylenebis (4-methyl-6-tert-butylphenol) (14.138 μg/ml), geniposide (11.331 μg/ml), 2-hydroxycinnamic acid (9.562 μg/ml), crotonoside (6.071 μg/ml), corymboside (4.511 μg/ml), apigenin-7-O-neohesperidoside (2.379 μg/ml), and naringenin (1.498 μg/ml). The most abundant phenolic acid in the extract was vanillyl alcohol (53.409 μg/ml), followed in order by 2,2′-methylenebis (4-methyl-6-tert-butylphenol) (37.218 μg/ml), 5-hydroxymethyl-2-furaldehyde (32.534 μg/ml), melittoside (21.282 μg/ml), *p*-coumaraldehyde (5.430 μg/ml), caffeic acid (3.655 μg/ml), isoferulic acid (2.377 μg/ml), 7-hydroxycoumarine (1.736 μg/ml), 4-hydroxybenzaldehyde (1.634 μg/ml), pyrogallol (1.036 μg/ml), and 2-hydroxycinnamic acid (0.691 μg/ml).

**TABLE 3:** Content of phenolic acids in sweet sorghum juice before and after polyphenol extraction.

Name	Formula	CAS_num	Rt [min]	Relative concentration (μg/mL)
Phenolic acids in sweet sorghum juice				
5′-S-Methyl-5′-thioadenosine	C11H15N5O3S	2,457-80-9	4.484	0.047 ± 0.003
4-Hydroxybenzaldehyde	C7H6O2	123-08-0	6.039	0.102 ± 0.002
7-Hydroxycoumarine	C9H6O3	93-35-6	9.791	0.137 ± 0.003
4-Hydroxybenzaldehyde	C7H6O2	123-08-0	1.208	0.835 ± 0.017
HOBT	C6H5N3O	2,592-95-2	6.959	1.083 ± 0.113
Naringenin	C15H12O5	480-41-1	9.777	1.498 ± 0.136
2′-O-Methyladenosine	C11H15N5O4	2,140-79-6	1.658	1.608 ± 0.159
Aflatoxin G2	C17H14O7	7,241-98-7	9.772	1.721 ± 0.173
Cytidine	C9H13N3O5	65-46-3	0.819	1.826 ± 0.098
2′-O-Methylguanosine	C11H15N5O5	2,140-71-8	2.041	1.907 ± 0.134
Apigenin-7-O-neohesperidoside	C27H30O14	17,306-46-6	6.939	2.379 ± 0.264
Coumaroyl Hexoside (isomer of 691, 692)	C15H18O8	7,139-64-2	5.36	4.042 ± 0.334
Corymboside	C26H28O14	73,543-87-0	6.003	4.511 ± 0.397
Crotonoside	C10H13N5O5	1818-71-9	1.297	6.071 ± 0.446
Vitexin	C21H20O10	3,681-93-4	6.606	7.216 ± 0.489
Hispidulin	C16H12O6	1,447-88-7	9.897	8.054 ± 0.887
2-Hydroxycinnamic acid	C9H8O3	614-60-8	1.209	9.56 ± 0.875
Genistein	C15H10O5	446-72-0	9.748	11.067 ± 0.569
geniposide	C17 H24 O10	24,512-63-8	5.439	11.331 ± 1.128
Isoferulic acid	C10 H10 O4	537-73-5	6.955	12.934 ± 1.364
2,2′-Methylenebis (4-methyl-6-tert-butylphenol)	C23H32O2	119-47-1	16.821	14.138 ± 1.783
Pseudouridine	C9H12N2O6	1,445-07-4	1.214	14.221 ± 1.778
Guanosine	C10H13N5O5	118-00-3	1.382	14.398 ± 1.651
p-Coumaraldehyde	C9H8O2	20,711-53-9	2.201	22.448 ± 1.983
4′,5,7-trihydroxy-3,6-dimethoxyflavone	C17H14O7	18,085-97-7	9.836	28.067 ± 2.215
Betaine	C5H11NO2	107-43-7	0.795	51.207 ± 3.124
D-(-)-Quinic acid	C7H12O6	77-95-2	0.865	53.112 ± 4.658
Phenolic acids in polyphenol extracts of Sweet Sorghum				
D-(-)-Quinic acid	C7H12O6	77-95-2	0.865	44.839 ± 3.321
Citric acid	C6H8O7	77-92-9	0.893	147.658 ± 6.648
Pyrogallol	C6H6O3	87-66-1	1.116	1.036 ± 0.125
2-Hydroxycinnamic acid	C9H8O3	614-60-8	1.209	0.691 ± 0.015
p-Coumaraldehyde	C9H8O2	20,711-53-9	2.201	5.430 ± 1.108
5-Hydroxymethyl-2-furaldehyde	C6H6O3	67-47-0	2.502	32.534 ± 1.118
NCGC00380682-01	C13H18O8	148707-37-3	5.502	2.472 ± 0.515
Caffeic acid	C9H8O4	331-39-5	5.72	3.655 ± 0.987
Melittoside	C21H32O15	19,467-03-9	5.749	21.282 ± 2.218
4-Hydroxybenzaldehyde	C7H6O2	123-08-0	6.039	1.634 ± 0.697
Isoferulic acid	C10H10O4	537-73-5	6.995	2.377 ± 0.315
Vanillyl alcohol	C8H10O3	498-00-0	7.313	53.409 ± 1.517
Protosappanin B	C16H16O6	102036-29-3	8.291	2.953 ± 0.548
Ethyl protocatechuate	C9H10O4	3,943-89-3	9.641	5.699 ± 0.981
7-Hydroxycoumarine	C9H6O3	93-35-6	9.791	1.736 ± 0.115
2,2′-Methylenebis (4-methyl-6-tert-butylphenol)	C23H32O2	119-47-1	16.821	37.218 ± 2.981

Phenol compound concentration μ g analyte/mL express. The data are mean ± standard deviation. Student t-test was used to determine the difference of statistical variables.

### 3.3 Antimicrobial Activity of Polyphenols From Sweet Sorghum Stalks

**FIGURE 5:**
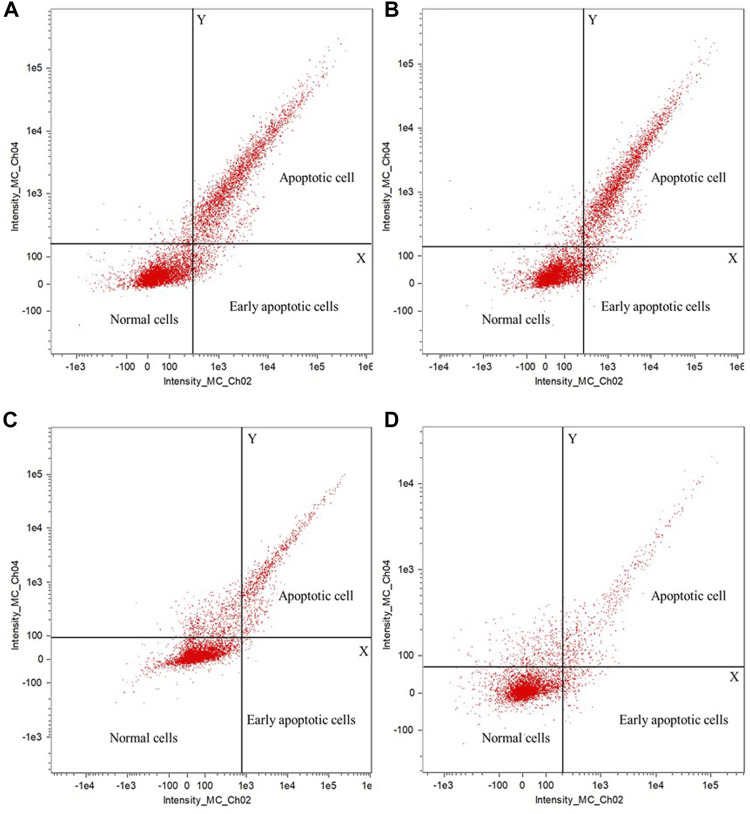
Effect of sweet sorghum polyphenols on apoptosis of pathogenic bacteria **(A)** is *Staphylococcus aureus* **(B)** is *Listeria* spp **(C)** is *Escherichia coli*, and **(D)** is *Salmonella* spp. In the coordinate system of the figure, the first quadrant contains apoptotic cells, the third quadrant contains normal cells, and the fourth quadrant contains early apoptosis cells.

The apoptotic effect of polyphenols from fresh sweet sorghum stems on the four foodborne pathogens was detected by flow cytometry. Figure 5 shows the test results for *S. aureus*, *Listeria* spp. *E. coli*, and *Salmonella* spp. (Figure 5A, B, C, and D, respectively). In the apoptosis test diagram, the upper right corner displays apoptotic cells, the lower right corner early apoptotic cells, and the lower left corner normal cells. After treating the four common foodborne pathogenic bacteria with 25 mg/ml sweet sorghum polyphenol extract, the number of early apoptotic and apoptotic cells consistently increased significantly. Sweet sorghum polyphenol extracts effectively inhibited the growth of all four microorganisms. The content of apoptotic cells were significantly higher for *S. aureus* and *Listeria* than for *E. coli* and *Salmonella* (Figure 5).

**FIGURE 6:**
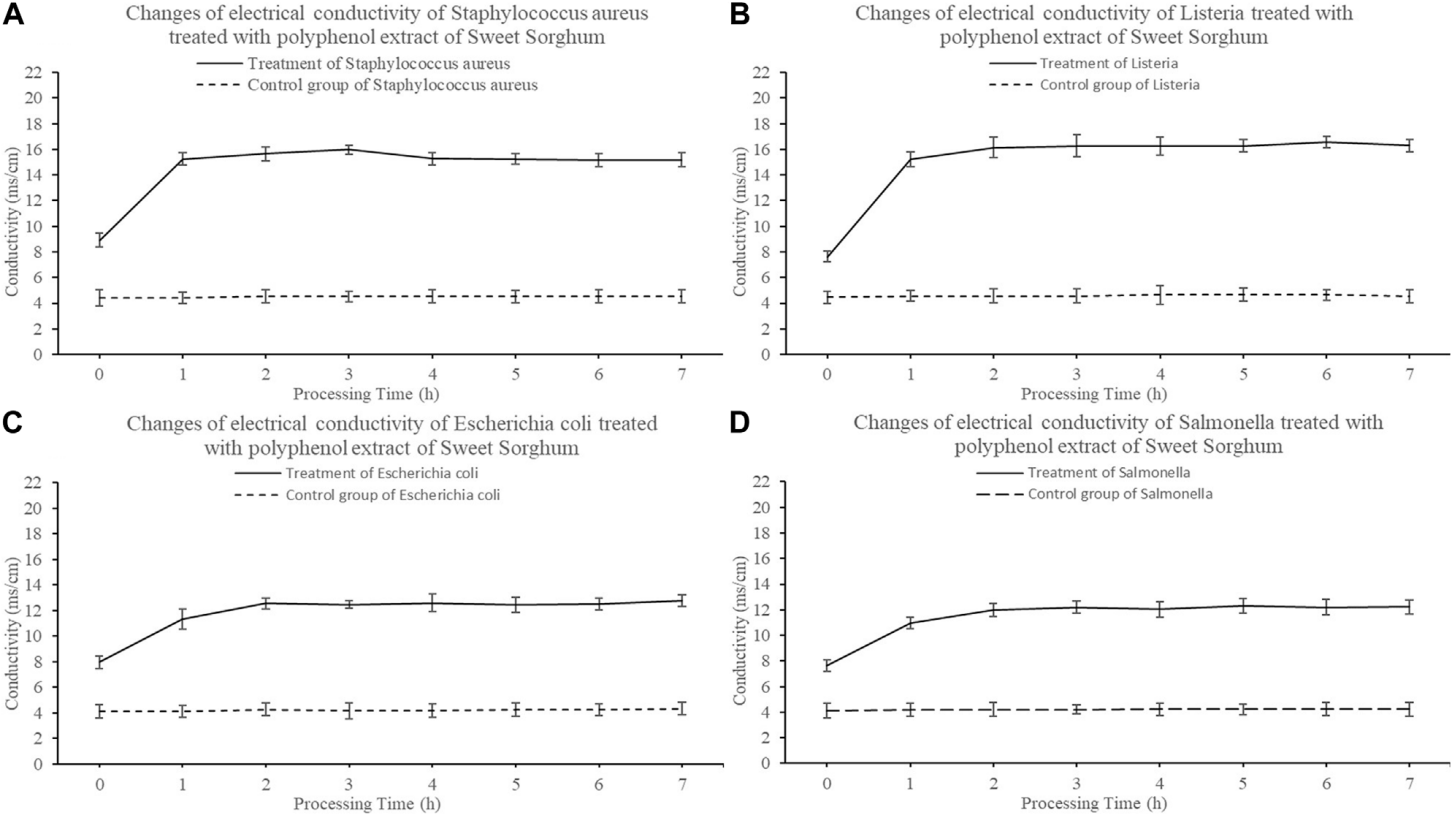
Effect of polyphenols from sweet sorghum on electrical conductivity of pathogenic bacteria **(A)** is *Staphylococcus aureus* **(B)** is *Listeria* spp **(C)** is *Escherichia coli* **(D)** is *Salmonella* spp. The data are expressed as mean ± standard deviation. One-way ANOVA (*p* < 0.001) and Dunnett’s posttest were used for statistical analysis.

The change in conductivity of the bacterial culture medium was measured. The electrical conductivity of the samples from strains exposed to sweet sorghum polyphenol extract was higher than that of the control and increased significantly in the first hour (*p* < 0.05) (Figure 6). The conductivity increased with the treatment time. *Staphylococcus aureus* increased from 8.92 m/cm to 15.18 m/cm (Figure 6A), *Listeria* increased from 7.63 m/cm to 16.30 m/cm (Figure 6B), *E. coli* increased from 7.96 m/cm to 12.771 m/cm (Figure 6C), and *Salmonella* increased from 7.654 m/cm to 12.234 m/cm (Figure 6D). The conductivity of each pathogen control group displayed almost no change, with an average value of 4.35 m/cm.

**FIGURE 7:**
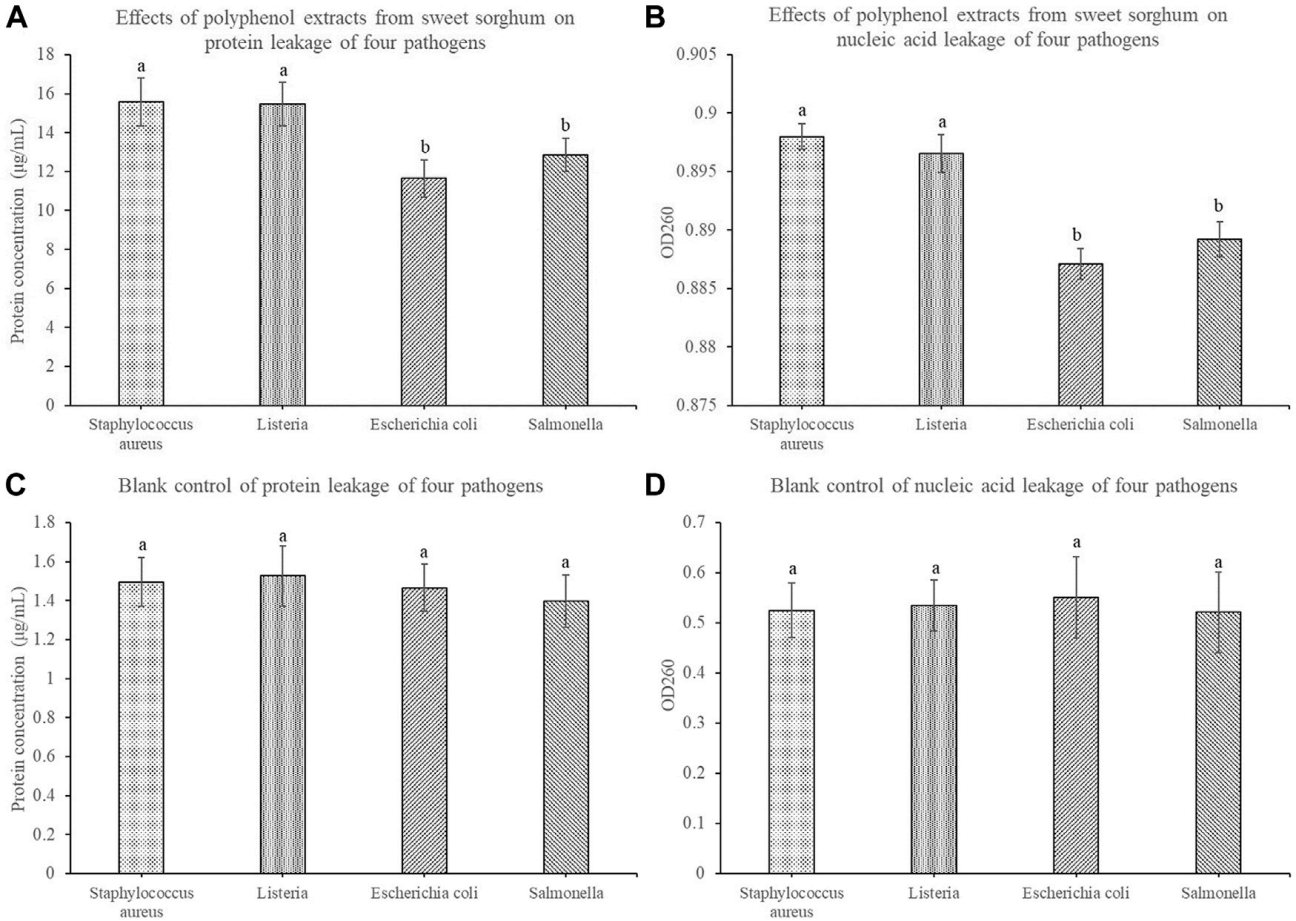
Effects of sweet sorghum polyphenol extract on protein leakage and nucleic acid leakage of four pathogenic bacteria **(A)** displays data for protein leakage and **(B)** displays data for nucleic acid leakage **(C)** and **(D)** display data of the corresponding blank control groups. The data are expressed as mean ± standard deviation. One-way ANOVA (*p* < 0.001) and Dunnett’s posttest were used for statistical analysis. Different lowercase letters in the figure indicate differences in significance.

To further explore the reasons for the increase in cell culture medium conductivity, we further measured the protein leakage and nucleic acid leakage of the four pathogens before and after treatment with sweet sorghum polyphenols. After treatment with sweet sorghum polyphenol extract, the protein leakage of *S. aureus* and *Listeria* was 15.56 μg/ml and 15.45 μg/ml, respectively. The protein leakage of *E. coli* and *Salmonella* was 11.64 μg/ml and 12.86 μg/ml, respectively (Figure 7A). The leakage of nucleic acids was also investigated. Among them, the nucleic acid leakage of *S. aureus*, *Listeria* spp. *E. coli*, and *Salmonella* spp. treated with sweet sorghum polyphenol extract was 0.898, 0.897, 0.887, and 0.889, respectively (Figure 7B).

## 4 Discussion

Vegetable food processing by-products are one of the main processing problems in the food industry. One of the main processing problems in this industry is the utilization of by-products. However, owing to their good technical or nutritional characteristics, they are also promising sources of compounds (Han et al., 2012). By-products in food processing, including waste, wastewater, seeds, peel, and rhizomes in the production and processing process, are good and effective sources of antioxidants (Patel, 2012).

The types and contents of phenolic acids are important indices to evaluate the utilization potential of plant polyphenol extracts (Scania and Chasani, 2021). However, the antibacterial activity of polyphenols may depend mainly on the concentration of polyphenols and the substitution position of phenolic hydroxyl groups on the aromatic ring. Phenolic acids in plants may eventually bind to proteins and polysaccharides through hydrogen bonds and hydrophobicity (Hammouda et al., 2012). Therefore, in the process of extracting phenolic acids, good solvents are needed to reflect high solubility and some metal ions are needed to destroy hydrogen bonds and increase the extraction rate. Generally, by-products in agricultural processing are the main sources of lignocellulose and phenolic acids (Monlau et al., 2015). For example, the grains after brewing beer are rich in ferulic acid and coumarin (Du et al., 2009). The by-products produced during onion and potato processing have anti-inflammatory effects (Albishi et al., 2013). The by-products of lettuce and chicory are rich in antioxidants and can be used in food (Kerdudo et al., 2014). There are a large number of phenolic acids in sugarcane and they have important effects on the color of fruit juice (Bucheli and Robinson, 1994). Furthermore, phenolic acids have good antioxidant capacity, inhibit the growth of bacteria, and kill tumor cells (Vaquero et al., 2007).

In the single factor experiment, when the concentration of ethanol in acid ethanol was 60%, the extraction rate of polyphenols from fresh sweet sorghum stems was the highest. This finding may be related to the solvent characteristics of ethanol as an organic solvent. Organic solvents have strong polarity, which can promote the dissolution of organic compounds, such as phenolic acids, to a certain extent. However, in the present study, with an increase in ethanol concentration in acid ethanol the extraction rate of polyphenols did not significantly improve. It is very likely that some substances with weak polarity, such as pigments and impurities, dissolve more in this process. This inhibits the complete dissolution of phenolic acid compounds (Gambuti et al., 2009). The optimal ethanol concentration determined in the present study was 60%.

When the solid-liquid ratio was 20:1 ml/g, the solubility of phenolic acids approached saturation. With a continuous increase in the solid-liquid ratio, the extraction rate of phenolic acids did not change significantly. This may be because the increase in ethanol solvent dissolves some impurities, such as pigment, and because of alcohol solubility (Zhang, 2010). Dissolution of effective components is prevented, so the ratio of liquid to material is 20:1 ml/g. The time of ultrasonic treatment in the extraction process was closely related to the dissolution of the solute. In this extraction process, the optimal ultrasonic treatment time was 60 min. A long treatment time may lead to the decomposition and transformation of polyphenols due to thermal effects. As well, if the ultrasonic time is too long, the polyphenols will be oxidized and decomposed, resulting in a decrease in the polyphenol extraction rate. Sixty minutes was determined to be the optimum ultrasonic time. The extraction rate of polyphenols from sweet sorghum reached a maximum at 40 °C. The increase in polyphenol extraction rate may be due to the strong diffusion and dissolution of polyphenols at a certain temperature (Wei, 1961). Higher temperatures may also destroy the structure of phenolic acids and aggravate the oxidative decomposition of polyphenols, resulting in a reduction in the extraction rate (Huo et al., 2017). The optimum ultrasonic temperature was determined to be 40°C.

The experimental data in Table 2 were fit and analyzed using the statistical analysis software Desig Expert 8. The quadratic multiple regression linear equation that was obtained was Y = 5.37 + 0.078A + 0.14B−0.005C−0.091D−0.21AB+0.20AC−0.15AD+0.14BC−0.068BD+0.26CD−0.58A2−0.67B2−0.79C2−0.71D2.

The analysis of the linear regression equation showed that F = 25.06, *p* < 0.0001. The mismatch difference was not significant (R^2^ = 0.962, R^2^ Adj = 0.923), indicating that the experimental model has a good fit and can effectively explain the changes in various values in the response surface test. The coefficient of variation was 3.82%, which is <10%, indicating that the experiment was highly accurate and reliable, with the same experimental results expected in repeat experiments.

The F value reflects the influence of each factor on the experiment. The sequence factors are treatment time, ethanol concentration, treatment temperature, and solid-liquid ratio. Among them, the primary term B, secondary term, and interactive term CD had significant effects on the extraction rate of polyphenols (*p* < 0.01). The interactive terms AB and AC were significantly different (*p* < 0.05).

Based on the optimization of the process of polyphenol extraction from fresh sweet sorghum stem by response surface methodology, it was found that the theoretical best extraction process was ethanol concentration 60.61%, liquid-solid ratio 20.51:1, ultrasonic time 60.02 min, and ultrasonic temperature 39.25°C. Under these conditions, the extraction rate of polyphenols from sweet sorghum was 5.39 mg/g. However, considering the convenience and efficiency in practical operation, the process parameters of polyphenol extraction were adjusted to an ethanol concentration of 61%, liquid-solid ratio of 21:1 ml/g, ultrasonic time of 60 min, and ultrasonic temperature of 40°C. The average extraction rate of polyphenols from sweet sorghum was 5.77 mg/g. The error was 1.5%. Therefore, the modified extraction process optimized by response surface methodology was stable and reliable.

To evaluate the bioactivity of polyphenols in fresh sweet sorghum stems, we measured the total phenol content and total antioxidant capacity before and after extraction. After the optimized extraction process, the total phenol content and total antioxidant capacity of fresh sweet sorghum stem extract were greater than those without extraction, which was also consistent with Qin et al. (2020) and Khan et al. (2018). The phenolic compounds extracted from green onion and chrysanthemum had higher 2,2-diphenyl-1-picrylhydrazyl scavenging ability and superoxide anion radical scavenging ability. The whole extraction process can enrich a large number of phenolic acids. Sweet sorghum, as a cheap cash crop, can also be an ideal material for the production of phenolic acids.

Fresh sweet sorghum juice is rich in phenolic acids, including polyphenols and flavonoids. These substances have good biological activities that include antioxidant, anti-inflammatory, and anti-tumor activities (Vanamal et al., 2017). In this study, sweet sorghum juice was rich in 31 polyphenols that included betaine, coumarin, guanosine, cinnamic acid, and naringenin. The polyphenol extract of sweet sorghum was rich in coumarin, vanillin, caffeic acid, coumaric aldehyde, cinnamic acid, and other 18 polyphenols. The main components of these extracts have a strong reducing ability. Zhao et al. (2015) described that extract from sugarcane was enriched in gallic acid, ferulic acid, coumarin, and chlorogenic acid, which have antioxidant and antibacterial properties. In addition, similar to the results of this study, Deseo et al. (2020) showed that the phenols in sugarcane molasses extract were closely related to antioxidant activity.

*Escherichia coli, S. aureus, Listeria* spp. and *Salmonella* spp. treated with 25 mg/ml sweet sorghum polyphenol extract were examined to determine the relationship between the antibacterial activity of the polyphenol extract and bacterial membrane permeability. The conductivity of the bacterial culture medium increased significantly in 1 h, but almost did not change between 1 and 7 h. At the same time, the electrical conductivity of the control group displayed almost no change within 7 h. This indicates that polyphenols extracted from sweet sorghum affect the integrity of bacterial membranes and cause electrolyte leakage. The test results of protein and nucleic acid content in the cell culture medium were consistent with the previous experimental results. Protein leakage and nucleic acid leakage were relatively low in the control group, and after treating four foodborne pathogenic bacteria with sweet sorghum polyphenol extract, it was found that the protein leakage and nucleic acid leakage in each treatment group increased significantly. The findings further indicate that the polyphenol extract from fresh sweet sorghum stem can increase the conductivity in bacterial culture medium in a short time, and its effect on gram-positive bacteria is significantly higher than the effect on gram-negative bacteria. The reason may be that polyphenols destroy the integrity of the bacterial cell membrane structure, resulting in the exudation of intracellular molecules, further resulting in an increase in the electrolyte content in the cell culture medium. This result is similar to that reported by Shen et al. (2015). The latter authors opined that cinnamaldehyde can destroy bacterial cell membranes. At the same time, with the increase in cinnamaldehyde concentration, the degree of damage of the cell membrane increased. Xu (2014) studied the effect of fennel seed extract on bacteria and found that an increase in treatment concentration and treatment time of polyphenol extract increased the permeability of the cell membrane. In the present study, the determination of cell apoptosis by flow cytometry confirmed the antibacterial effect of sweet sorghum polyphenol extract on foodborne pathogens. After treatment of the four foodborne pathogens with fresh sweet sorghum stem polyphenols, the number of apoptotic cells exceeded the numbers in the control group of each bacterium.

In general, the position of phenolic hydroxyl groups in phenols has a significant impact on the antioxidant and antibacterial capacities of polyphenols (Toshitsugu et al., 2004). For example, cinnamic acid can lead to irreversible changes in membrane structure, so as to release intracellular components out of cells (Borges et al., 2013). This may be because polyphenols change the hydrophobicity of the membrane structure, resulting in local rupture of membrane structure and formation of holes. The antibacterial effect of flavonoids is different from that of polyphenols (McRae et al., 2007). It may form a complex with the components of the bacterial cell wall, resulting in a gap in the cell wall. The antibacterial mechanism of some substances involves the inhibition of DNA helicase (Matja et al., 2010), resulting in the failure of membrane structure to inhibit bacterial growth. In this study, the main antibacterial mechanism of fresh sweet sorghum stem polyphenols appears to be polyphenol-mediated destruction of the integrity of the cell membrane, resulting in cell apoptosis. Polyphenols may also form a complex with essential substrates in bacterial metabolism, resulting in bacterial metabolic disorder and inactivation of protein variability. These mechanisms require further study.

In conclusion, fresh sweet sorghum stem extract is rich in phenolic compounds, has good antioxidant capacity, and has potent antibacterial action for common foodborne pathogenic bacteria. Its antibacterial mechanism involves the toxicity of polyphenols, particularly the destruction of the microbial membrane structure. Therefore, sweet sorghum polyphenol extract can be used as a natural bioactive material with high added value for antibacterial applications in the food industry.

## Data Availability

The original contributions presented in the study are included in the article/Supplementary Materials, further inquiries can be directed to the corresponding author.
